# An Unusual Case of Cardiac Mass: A Multimodal Approach in Diagnosis and Treatment

**DOI:** 10.3390/healthcare12101009

**Published:** 2024-05-14

**Authors:** Ljiljana Rankovic-Nicic, Milica Dragicevic-Antonic, Zelimir Antonic, Vladimir Mihajlovic, Masa Petrovic, Tjasa Ivosevic, Gordana Stamenkovic, Svetislav Pelemis, Milovan Bojic

**Affiliations:** 1 Institute for Cardiovascular Diseases “Dedinje”, 11040 Belgrade, Serbia; 2 Faculty of Medicine, University of Belgrade, 11000 Belgrade, Serbia

**Keywords:** intracardiac mass, left atrial thrombi, CMR, multimodal imaging

## Abstract

Diagnosing intracardiac masses poses a complex, multimodal challenge. We present the case of a 72-year-old woman with a history of rheumatic fever leading to mitral stenosis and a previous mitral valve commissurotomy who reported fatigue, weakness, and palpitations over the past three months. Echocardiography revealed a tumor (53 × 40 mm) in the enlarged left atrium, attached by a wide base to the left atrium wall, exhibiting variable densities. Computerized tomography identified a heterodense mass (53 × 46 × 37 mm) with similar attachments. Angiography showed two branches from the circumflex artery intricately associated with the mass. Despite unsuccessful embolization of the mass’ blood supply, surgical intervention including mitral valve replacement, tricuspid valve annuloplasty, and tumor removal was pursued. Pathohistological analysis confirmed the mass as a thrombus. During the postoperative follow-up, the patient presented with no complaints. Follow-up echocardiography indicated the normal function of the mechanical mitral valve prosthesis and the absence of intracardiac masses. While it remains unknown whether this neovascularization is specific to patients with severe mitral valve disease, this case highlights the diagnostic challenges of differentiating between thrombi and tumors in the context of mitral valve disease. It illustrates the critical role of multimodal imaging in elucidating the anatomical and functional relationships within the heart, thereby guiding accurate diagnosis and effective treatment.

## 1. Introduction

The diagnosis of an intracardiac mass represents a clinical challenge requiring multimodal diagnostic approaches. Differential diagnosis encompasses a wide range, including thrombi, vegetations, implanted devices, coumadin ridge, Chiari network, Eustachian valve, crista terminalis, and tumors. Some may be classified as anatomical variations, while others may indicate differing prognoses. Therefore, accurate and early diagnosis is essential to subsequently facilitate the appropriate assessment of treatment strategy. Primary tumors of the heart are rare, with three-quarters being benign. The most common primary cardiac tumor is myxoma, often diagnosed based on the morphology and attachment site of the mass to the heart. Given that myxomas can mimic various cardiovascular diseases and are typically detected in symptomatic patients, a high index of clinical suspicion is crucial for their early and accurate diagnosis [1,2].

On the other hand, cardiac thrombi tend to appear more frequently than cardiac myxomas and are typically located in the left ventricle (66.8% of cases). Less frequently, they are also found in the left atrium (LA) (32% of cases) and generally occur in patients with organic heart disease such as mitral valve disease, atrial fibrillation, ischemic heart disease, dilated cardiomyopathy, and in patients with a previous history of heart valve surgery. The formation of a thrombus involves a complex interplay of hypercoagulability, hemodynamic changes, and endothelial injury—often referred to as Virchow’s triad. In some cases, an atrial thrombus may be pedunculated which can be misdiagnosed as a myxoma, which can lead to unnecessary surgical resection. Notably, in cases of LA myxoma with mitral stenosis, it can be particularly difficult to differentiate a thrombus from a myxoma [1,2,3].

The gold standard for diagnosing an intracardiac mass is a biopsy. However, due to its invasiveness and associated risks, biopsies are not always feasible, thus further complicating the diagnostic process. This is especially true when masses present unusual shapes and localizations.

We report a distinctive case of a cardiac mass, highlighting the value of multimodal imaging and a collaborative team approach in diagnosing and managing complex intracardiac masses.

## 2. Case Presentation

A 72-year-old woman was presented to the department with a 3-month history of fatigue, weakness, and palpitations. Her past medical history included rheumatic fever when she was 13 years old, causing mitral stenosis to develop later in life, a mitral valve commissurotomy performed in 1984, and a twenty-year-long history of atrial fibrillation. The patient continued taking acetylsalicylic acid (100 mg per day) regularly for 19 years after her surgery but discontinued after an episode of transient ischemic attack (TIA) in 2003 when warfarin was also introduced to her therapeutic regime. It is important to note that at the time of admission, it was not known whether the patient consistently maintained an International Normalized Ratio above 2.0. This uncertainty is partly attributable to the patient’s lack of regular follow-ups.

**Figure 1:**
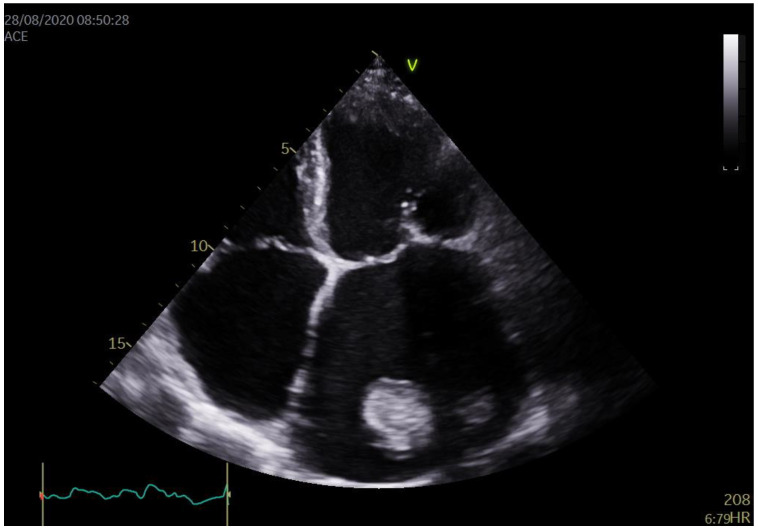
Echocardiography. Tumor formation in the enlarged left atrium (LA) (70 mm). One of the dimensions measured 53 × 40 mm, fixated by a wide base to the free wall of the LA.

On admission, a basic blood panel was performed which was unremarkable. The electrocardiogram revealed atrial fibrillation with a heart rate of 70 beats per minute and signs of initial left ventricular hypertrophy. The patient’s blood pressure was within normal limits (125/75 mmHg). Given the anamnestic information regarding a TIA, a carotid Doppler was performed, which demonstrated stenosis of both carotid arteries (35–40%). Neurological findings were unremarkable. An echocardiogram revealed a tumor in the enlarged LA measuring 70 mm with varying densities, notably 53 × 40 mm in one dimension, fixated by a wide base to the free wall of the LA (Figure 1). The mass was immobile and limited as it did not continue into the left auricle. The mitral cusps were fibrously altered, thickened, and exhibited reduced mobility, while the posterior mitral cusps were immobile. Doppler showed a pressure gradient measuring 19/11 mmHg and MVA/PHT/1.4 cm^2^. The mitral orifice area was 1.0 cm^2^, planimetrically measured. Mild mitral insufficiency, severe tricuspid insufficiency with medium pressure in the right ventricle of 74 mmHg, and regular dimensions of the left ventricle (53/33 mm) without a drop in kinetics, ejection fraction (EF) 50% was noted. Cardiomagnetic resonance (CMR) was initially suggested; however, at the time, it was not available at our institution, and therefore a cardiac computerized tomography (CT) was performed instead.

**Figure 2:**
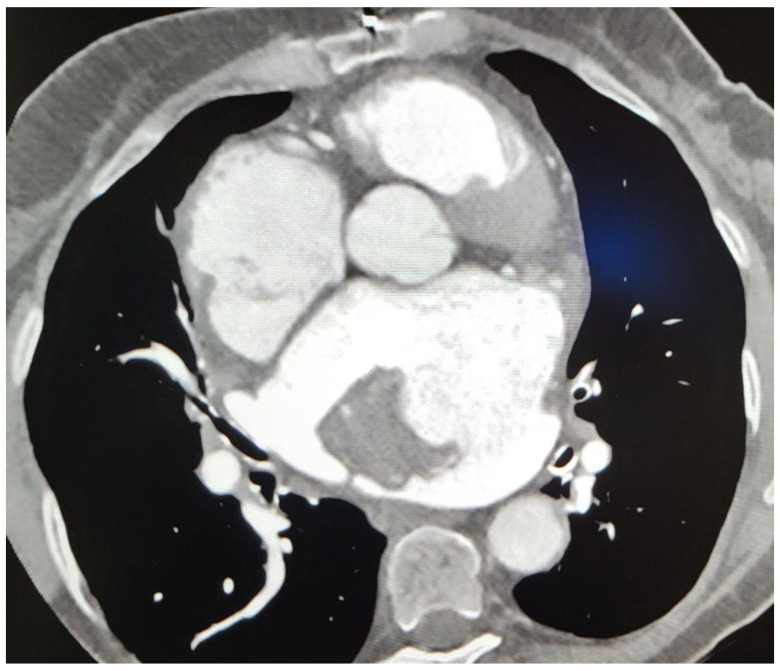
Computerized tomography (CT). Sharply limited heterodense change with a diameter of 53 × 46 × 37 mm and a wide base attached to the wall of the atrium.

**Figure 3:**
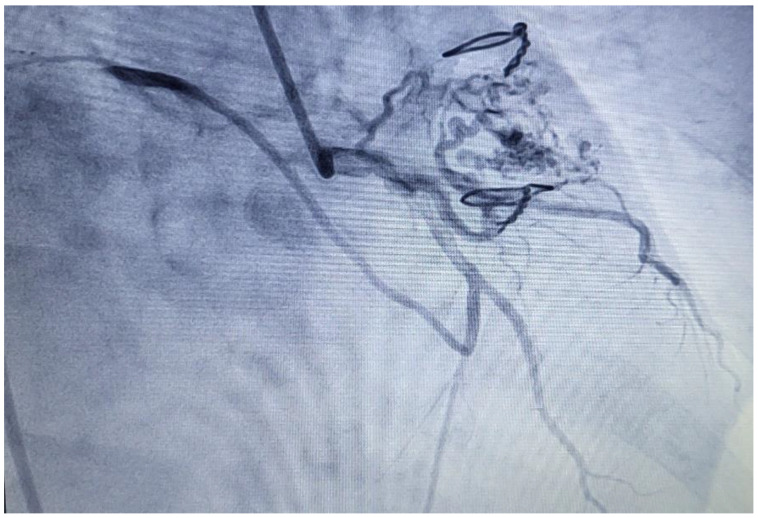
Coronoragraphy. Circumflex artery (Cx), and two branches. The first branch formed a loop that communicated with the auricle of the left atrium (LA), and the second branch separated at an angle and ran along the back wall of the LA with communication with the structure in LA.

Subsequent CT imaging provided further details, including the mass’s heterodense nature and its vascular connections, raising questions about its origin. On CT, a sharply limited heterodense change with a diameter measuring 53 × 46 × 37 mm with a wide base attached to the wall of the LA was observed (Figure 2). Two branches from the proximal part of the circumflex artery (Cx) were observed, with the first forming a loop that communicated with the auricle of the LA, and the second branch separating at an angle running along the posterior wall of the LA with communication with the structure in the LA (Figure 3). The vascularized mass raised questions regarding its origin, warranting further investigation. Given the diagnosis of moderate to severe mitral stenosis and findings from the cardiac CT that indicated that the right ventricle is in close proximity to the lower third of the sternum and the xiphoid process, the cardio-surgical team opted for a less invasive approach. Considering that this would be a redo operation for the patients, the team decided to first attempt embolization of the lesion to cut off its blood supply, thereby inducing tissue necrosis and subsequent reduction in tissue volume.

Embolization of the communicating blood vessels was attempted but failed due to the unfavorable anatomy of the artery. Subsequently, surgical treatment was recommended, and the patient underwent mitral valve replacement with a mechanical prosthesis (Onyx 31–33), tricuspid valve annuloplasty (Medtronic 32), and removal of the tumor mass from the LA. The procedure was performed through a medial sternotomy with general heparinization. No mass was found in the right atrium. The extirpated mass was sent for pathohistological analysis which showed that the mass consisted of acellular tissue predominantly composed of fibrin, which formed Zahn’s lines. The tissue matrix was interspersed with rare apoptotic cells, including neutrophil granulocytes, foamy macrophages, and lymphocytes. Additionally, dystrophic calcifications were also observed in some areas. These morphological changes correspond to those typically found in a thrombus.

The patient’s postoperative course was marked with complications, including increased drainage noted on day 0, necessitating a return to the operating room for hemostatic revision, where the specific site of bleeding was identified. Subsequently, the patient remained hemodynamically stable. On the second postoperative day, anticoagulant therapy was initiated, starting with low molecular weight heparin and later transitioning to warfarin by the fourth postoperative day. A control cardiac ultrasound confirmed the proper function of the artificial mitral valve, with gradients measured at 10/5 mmHg, trace tricuspid regurgitation (TR1+), and an LA clear of any mass. The ejection fraction was 40%. The patient was discharged on the sixth postoperative day.

At the one-month post-op follow-up visit, the patient presented without any complaints. The complete blood panel was normal, and the patient continued regular anticoagulant therapy and warfarin to maintain INR over 2.5. The normal function of the mechanical mitral prosthesis was noted on the echocardiogram, and a mean gradient of 6 mmHg was measured via Doppler without paravalvular mitral regurgitation (MR). On examination, the left ventricle had an overall reduced systolic function (EF = 40%), and in the tricuspid position, an annulus with minor tricuspid insufficiency, and medium pressure in the right ventricle of 53 mmHg was observed. Further evaluation using CMR and consultation with a hematologist was recommended to explore the possibility that the thrombus was caused by a hematological disease and an inadequate response to anticoagulant therapy. However, the patient declined further evaluations due to the absence of symptoms and the overall stability of their clinical presentation.

## 3. Discussion

The differential diagnosis of intracardiac masses remains a formidable clinical challenge. Typically, the initial diagnosis is based on a comprehensive, non-invasive diagnostic approach, necessitated by the technical complexities and inherent risks associated with biopsy procedures, despite the latter being considered the diagnostic gold standard. Among the array of cardiac imaging modalities, echocardiography is often the first-line choice. However, imaging modalities like cardiac CT and, especially, CMR, play critical roles when echocardiographic results are inconclusive or technically limited. It is imperative that imaging findings are interpreted within the relevant clinical context to mitigate the risk of misdiagnosis, particularly in cases with complicating factors such as infective endocarditis or thrombus formation [3,4,5,6].

Contrast echocardiographic perfusion imaging has emerged as a promising diagnostic tool adept at differentiating the neovascularization typical of malignancies from the non-vascular nature of thrombi and the inconsistent vascular features of stromal tumors. In their research, Kirkpatrick et al. included a cohort of sixteen patients with cardiac masses, who were assessed using power-modulation imaging subsequent to the administration of echocardiographic intravenous contrast. This involved both visual and software-based analysis of pixel intensities within the mass and a neighboring myocardial area. Each mass was confirmed through pathological diagnosis or resolved with anticoagulation treatment. Findings from this series indicate that echocardiographic contrast perfusion imaging is effective in discerning between different types of cardiac masses. Notably, malignant and vascular tumors exhibited greater enhancement when compared to the surrounding myocardium, while stromal tumors and thrombi were less enhanced [7].

The clinical presentation of cardiac tumors is highly variable and dependent upon several factors such as size, location, relation to other surrounding structures, and mobility. CMR, on the other hand, further enhances diagnostic accuracy by providing non-invasive, multiplanar insights into the lesion’s anatomical relationships and tissue characteristics, thereby facilitating the differentiation between pseudomasses and true masses, as well as differentiating between benign and malignant lesions. During CMR examination, different sequences enable the better structural characterization of the tissue. Specifically, perfusion sequences play a crucial role in evaluating the vascularization of masses. Additionally, early gadolinium enhancement (EGE) and late gadolinium enhancement (LGE) sequences are pivotal for identifying thrombi and further characterizing the mass. “Black-blood” imaging can also be employed to pinpoint the location of a potential cardiac mass and to gather more detailed information regarding its tissue composition [8]. Castro-Martín et al. presented a case series including four cases of intracardiac masses where CMR stands out as the most valuable non-invasive technique as it provides more clinical information, which in turn plays a further role in influencing the trajectory of further therapy. Although there are some technical limitations and no single characteristic can definitively establish a diagnosis, the probability of accurately diagnosing conditions continues to improve daily. In every instance within this case series, CMR positively influenced management decisions. The integration of clinical and epidemiological data with sophisticated imaging technologies enhances diagnostic precision while avoiding the need for unnecessary invasive procedures [9].

As presented in the literature, myxomas are the most prevalent primary cardiac tumors, typically manifesting symptoms like exertional dyspnea, fever, weight loss, syncope, sudden death, and hemoptysis. The size of myxomas can range significantly, and they are predominantly found originating in the LA [1,2]. Despite echocardiography’s role as the diagnostic modality of choice for myxoma, differentiating between a mass, thrombus, and tumor in the LA with preoperative transthoracic or transesophageal echocardiography presents a unique challenge, especially in patients with mitral valve disease and chronic atrial fibrillation. In this group of patients, considering an initial strategy of systemic anticoagulation followed by repeat imaging could help to prevent unwarranted surgical interventions. However, the safety of this approach remains unconfirmed [10,11].

The formation of left atrial thrombi (LAT) is recognized as a well-known complication of atrial fibrillation and rheumatic mitral valve disease, with a tendency to form particularly in patients with atrial fibrillation, mitral valve disease, and other notable conditions due to relative stasis in the LA’s appendage. The prevalence of cardiac thrombi in patients with atrial fibrillation or left ventricular dysfunction has been reported to range from 2–7%. Thrombi pose a significant risk for systemic thromboembolism and, less frequently, mechanical valve obstruction. Thus, further emphasizing the importance of their early detection and management, requiring a multidisciplinary approach to the management of these patients. Typically, giant thrombi that develop inside the atrial chamber are immobile, well-organized, fibrotic, and in close relation to the atrial wall. Atrial thrombi often masquerade as myxomas and have been reported in the literature. Mahmoud et al. conducted a PubMed search on cases where LATs were incorrectly diagnosed as myxomas, identifying 24 instances. Of these, 20 patients exhibited procoagulant conditions: 11 with atrial fibrillation, five with mitral stenosis, three with left ventricular dysfunction, and one with systemic lupus erythematosus. Information was lacking for three patients, and another had previously undergone cardiac transplantation. The precise differentiation between LATs and myxomas in patients predisposed to procoagulant conditions may not be feasible without a tissue biopsy. Given their poor response to thrombolytic therapy and the associated risk of systemic embolization, in the rare instances of giant LATs, surgical removal is considered to be the treatment of choice [10,11,12].

Interestingly, the occurrence of neovascularity and fistula formation from the coronary arteries to the LA, as documented in both retrospective studies and case reports, highlights a complex aspect of atrial thrombosis in patients with mitral valve disease [13,14,15,16,17,18]. The precise mechanism underlying fistular formation and neovascularization remains unclear; however, numerous studies have suggested that neovascularity and the development of fistulas from the coronary arteries to the LA are commonly associated with organized atrial thrombosis in patients afflicted with mitral valve disease. In a retrospective analysis of 507 patients, neovascularization in the LA, identified through coronary angiography, was present in 30 out of 76 patients with LAT. All of these cases of neovascularization, along with fistula formation, originated from the circumflex coronary artery without any evidence of atherosclerotic lesions. Surgical findings confirmed atrial thrombi in 25 of these patients [13]. The study by Russo et al. pointed out that arteriographic evidence of neovascularity and fistula connections between coronary arteries and the LA have occasionally been observed in association with LA thrombosis among patients with mitral valve disease. The relevance of these arteriographic findings for diagnosing atrial thrombi was assessed in 112 patients with mitral valve disease, with surgical comparisons also drawn [14]. While these angiographic observations support the diagnosis of atrial thrombosis, their detection during coronary arteriography in patients with mitral valve disease is considered beneficial. Such findings could enhance the accuracy of diagnosing thrombosis, particularly in patients who have not experienced prior embolic events or in cases where echocardiography did not detect thrombi. Al-Bezem et al. described a case where angiography confirmed multiple coronary neovascularizations originating from the left circumflex artery (LCx) extending towards multiple thrombi in the LA of a patient with severe mitral stenosis. Following four weeks of anticoagulation therapy with warfarin, the authors reported near-complete resolution of both the thrombi and neovascularizations [16]. Similarly, Serafino et al. described the case of a 57-year-old female patient with moderate rheumatic mitral stenosis who was not on an anticoagulation regimen for atrial fibrillation. The patient had a large, organized mass in the LA. In this case, the left atrial mass was related to mitral stenosis with atrial fibrillation, without anticoagulation, and increased LA. Initially, this was diagnosed as a thrombus on echocardiogram. However, during the preoperative catheterization, the vascularized mass created doubts as to the nature of the mass. The mass has extensive vascularization sourced from both the left and right coronary arteries. Ultimately, the patient underwent surgical removal of the mass, which was subsequently confirmed as a giant LAT [19]. Sakamoto et al., present a study that included 34 patients in whom coronary angiography showed coronary neovascularity in the LA with coronary artery-left atrial fistulas indicative of a thrombus in the LA appendage [20]. In this study, transthoracic echocardiography was performed on all 34 patients within a week of undergoing coronary angiography. Subsequently, open-heart surgery was carried out on 28 patients between 2 and 31 months after the angiography. In 28 patients, coronary neovascularity and a coronary artery-left atrial fistula originating from the LCx were identified. In the remaining six patients, the fistulas arose from both the LCx and the right coronary artery. Echocardiographic detection of a left atrial appendage thrombus was confirmed in only one of the 34 patients included in the study. However, during open-heart surgery, a left atrial appendage thrombus was found in 18 of the 28 operated patients and was subsequently resolved. The conventional therapeutic strategy for cardiac masses has typically involved surgical resection. For right-sided cardiac masses, the AngioJet device offers an alternative treatment option for patients who are either reluctant or contraindicated to receive thrombolysis or undergo surgical embolectomy [21]. Conversely, Valentin et al. presented a case in which coil embolization of tumor-related coronary arteries in the LA successfully interrupted the coronary supply to a cardiac metastasis originating from a uterine leiomyosarcoma [22].

These phenomena, often arising from the circumflex coronary artery without accompanying atherosclerotic lesions, suggest a unique pathophysiological mechanism, the specifics of which remain yet to be resolved. Whether this neovascularity is exclusive to patients with severe mitral valve disease is an open question, as current literature does not report such findings in patients without mitral valve disease. The accurate diagnosis of intracardiac masses involves a nuanced understanding of a variety of diagnostic tools and a careful consideration of the patient’s clinical context. The evolution of imaging technologies continues to enhance our diagnostic capabilities, yet the complexity of differentiating between various types of cardiac masses emphasizes the necessity for a multidisciplinary approach to patient care.

## 4. Conclusions

The differentiation between LATs and tumors in patients predisposed to procoagulant conditions presents significant challenges. In the presented case, an enlarged LA harbored a mass whose nature was obscured by vascularization from the coronary arteries, complicating its preoperative diagnosis. The presence of a vascularized mass introduced uncertainties, making it difficult to ascertain its origin. The effective diagnosis and management of such cases necessitate a collaborative approach among heart team members, emphasizing the value of discussion and collective decision-making. Moreover, the application of multimodal imaging techniques plays a crucial role in enhancing our understanding of the anatomical and functional relationships between cardiac structures and the intracardiac mass. This, in turn, aids in achieving a more accurate diagnosis and formulating an appropriate treatment strategy.

## Data Availability

Data are contained within the article.
